# Real world evidence reveals improved survival outcomes in biliary tract cancer through molecular matched targeted treatment

**DOI:** 10.1038/s41598-023-42083-4

**Published:** 2023-09-18

**Authors:** Bernhard Doleschal, Hossein Taghizadeh, Gerald Webersinke, Gudrun Piringer, Georg Schreil, Jörn Decker, Karl J. Aichberger, Patrick Kirchweger, Josef Thaler, Andreas Petzer, Clemens A. Schmitt, Gerald W. Prager, Holger Rumpold

**Affiliations:** 1 Department of Internal Medicine I for Hematology with Stem Cell Transplantation, Hemostaseology, and Medical Oncology, Ordensklinikum Linz, Seilerstaette 4, 4010 Linz, Austria; 2 Department of Internal Medicine, University Hospital St. Pölten, St. Pölten, Austria; 3 Laboratory for Molecular Genetic Diagnostics, Ordensklinikum Linz, Linz, Austria; 4 Department of Oncology and Hematology, Kepler University Clinic Linz, Linz, Austria; 5 Medical Faculty, Johannes Kepler University Linz, Linz, Austria; 6 Department of Internal Medicine, State Hospital Pyhrn Eisenwurzen, Steyr, Austria; 7 Department of Internal Medicine, State Hospital Rohrbach, Rohrbach, Austria; 8 Department of General and Visceral Surgery, Ordensklinikum Linz, Linz, Austria; 9 Gastrointestinal Cancer Center, Ordensklinikum Linz, Linz, Austria; 10 Department of Internal Medicine IV, Hospital Wels-Grieskirchen, Wels, Austria; 11 Division of Oncology, Department of Medicine I, Medical University Vienna, Vienna, Austria

**Keywords:** Cancer, Drug discovery, Biomarkers, Gastroenterology, Health care, Medical research, Molecular medicine, Oncology

## Abstract

Biliary tract cancers are rare cancers with poor prognosis due to a lack of therapeutic options, especially after the failure of first-line systemic treatment. Targeted treatments for this clinical situation are promising and have entered clinical practice. We aimed to describe the overall survival of matched targeted treatment after first-line treatment in patients with biliary tract cancers in an Austrian real-world multicenter cohort. We performed a multicenter retrospective chart review of patients with biliary tract cancer between September 2015 and January 2022. Data, including comprehensive molecular characteristics—next generation sequencing (NGS) and immunohistochemistry (IHC), clinical history, surgical procedures, ablative treatments, patient history, and systemic chemotherapy, were extracted from the records of the participating institutions. Targeted treatment was matched according to the ESMO scale for the clinical actionability of molecular targets (ESCAT). We identified 159 patients with the available molecular characteristics. A total of 79 patients underwent second-line treatment. Of these, 36 patients received matched targeted treatment beyond the first-line and were compared with 43 patients treated with cytotoxic chemotherapy in terms of efficacy outcomes. For Tier I/II alterations, we observed a progression free survival ratio (PFS_targeted_/PFS_pre-chemotherapy_) of 1.86, p = 0.059. The overall survival for patients receiving at least two lines of systemic treatment significantly favored the targeted approach, with an overall survival of 22.3 months (95% CI 14.7–29.3) vs. 17.5 months (95% CI 1.7–19.8; p = 0.048). Our results underscore the value of targeted treatment approaches based on extended molecular characterization of biliary tract cancer to improve clinical outcomes.

## Introduction

Biliary tract cancers (BTC) comprise a heterogeneous group of rare tumors with a poor prognosis. The incidence in Western countries is rising, especially for the intrahepatic subtype, which may be linked to metabolic-associated conditions such as obesity, type-2 diabetes, or non-alcoholic liver disease^1,2^. Overall survival (OS) is still poor, with a 5-year OS rate of < 20% across all subtypes^1,3,4^.

Recently, a new first-line standard incorporating checkpoint inhibitors (CPI) in a first-line metastatic setting has emerged^5^. Treatment after the first-line predominantly comprises cytotoxic chemotherapy. However, the proof of its effectiveness is rather weak, depending on the retrospective analyses and two prospective randomized trials^6–8^. The advent of sophisticated molecular techniques has paved the way for precision medicine in BTCs. It is estimated that up to 50% of BTCs are potential candidates for molecular-informed therapy^9,10^. Several smaller phase I/II trials, basket trials, and case series show the potential of targeted treatment regimens in second-line and beyond^11–14^. Currently, testing for fibroblast growth factor receptor 2 (*FGFR2)* or neurotrophic tropomyosin kinase receptors (*NTRK)* fusion, isocitrate dehydrogenase 1 *IDH1* or B-rapidly accelerated fibrosarcoma kinase (*BRAF)* hotspot mutations, and human epidermal growth factor receptor 2 (*HER2/neu)* amplification or microsatellite status is strongly recommended by the new BTC ESMO guidelines^4^. Tackling other pathogenic variants found in next-generation sequencing (NGS) reports seems of less value.

Whether the strategy of comprehensive molecular profiling—NGS, immunohistochemistry (IHC)—of all BTCs, at least in the second-line setting, is efficient in terms of outcome parameters in real-world conditions remains unclear. Although retrospective series suggest a clinical benefit of molecular profiling, these analyses are hampered by the local availability of targeted treatments, advances in molecular techniques that expand potential molecular vulnerabilities, and ethnic differences in the molecular profile of BTC^11,13^.

Therefore, the aim of this study was to assess the efficacy of matched targeted treatment in second-line and beyond therapy in patients with BTC in an Austrian real-world multicenter cohort.

## Materials and methods

### Patients

In this trial, we conducted a retrospective chart review of patients diagnosed with BTC at five cancer centers in Austria between September 2015 and January 2022.

Patients were included in the study if they had a histologically proven diagnosis of BTC and molecular profiling of tumor tissue. Decisions for individual molecular-matched therapeutic options were based on current scientific and clinical experience, clinical trial availability, and approved agents at the time.

Data collected from the patients’ electronic records included demographics, clinical history, surgery, local therapeutic approaches, chemotherapy, targeted therapies, response to targeted treatment, and survival status as of September 2022. Stages were defined according to the TNM 8th edition.

### Statement of ethics

Written informed consent for molecular analysis was obtained from all patients during the routine clinical workflow, and the study protocol was approved by the local committees on human research (Ethics Committee Land Oberoesterreich, 1100/2023 and Ethics Committee of the City of Vienna, EK-1099/2021), ensuring that it conformed to the ethical guidelines of the 1975 Declaration of Helsinki.

The datasets used and/or analysed during the current study are available from the corresponding author on reasonable request.

### Molecular profiling and classification of mutations

Genetic variants were considered for statistical analysis according to criteria as stated by Cao et al.^15^.

We tested two variant classification systems in precision oncology that are typically used in molecular tumor boards. ESCAT, as a framework for the actionability of molecular targets^16^, and the National Center for Tumor Diseases (NCT) Heidelberg variant classifier were used to rank the clinical evidence for the matched targeted therapies^17–19^. Sample collection and NGS molecular profiling were performed using tumor tissues obtained from core tumor biopsies or surgical samples. DNA was analyzed using customized NGS panels: the TruSight Tumor 170—Illumina hybrid capture platform (170 genes for mutations) amplifications or fusions) or the FoundationOne CDx hybrid-capture NGS service platform (324 genes for both mutations and fusions) Foundation Medicine. Inc). Additionally, data on defective mismatch repair (dMMR)/microsatellite instability (MSI) status, PD-1 or PDL-1 and *HER2neu* or *EGFR* IHC were collected when reported.

### Study end points

Our primary objective was to assess the overall survival (OS) of patients with ESCAT I-IV or NCT m1-m4 alterations matched to targeted drugs compared with those treated with chemotherapy. OS was defined as the time from the starting date of first-line systemic chemotherapy to the date of death; patients without documented death on the cut-off date were censored on the date the patient was last known to be alive.

The secondary objectives were to assess (i) the time from progression or failure of first-line treatment to death or last follow-up (OS@ 2nd line) and (ii) determine the overall response rate (ORR).

Time to failure of strategy (TFS) was defined as the time from the start of the first-line chemotherapy to the start of the second-line treatment (matched targeted treatment or non-targeted therapy equivalent to chemotherapy). PFS was defined as the time from the start of chemotherapy or matched targeted treatment to the date of the first documentation of disease progression or death from any cause, whichever occurred. The activity of targeted treatment was further described using the PFS ratio, defined as PFS achieved on matched targeted treatment divided by PFS or TFS observed on previous therapy.

We used the modified PFS (mPFS) approach proposed by Mock et al.^20^. An mPFS ratio of ≥ 1.3 was considered beneficial based on the considerations of Bailey et al. and Von Hoff et al. using each patient as his or her individual control, which is a study endpoint for precision medicine studies as recommended by health agencies such as the European Medicines Agency^21,22^. Survival data derived from statistical analysis is expressed as median values.

### Statistics

Descriptive statistics were used to summarize the baseline data of all patients with the molecular test results. Logistic regression was used to estimate the odds ratio for receiving targeted treatment according to the ESCAT or NCT classification of alterations.

Survival analysis was conducted for patients who received at least first-line palliative chemotherapy, and further analysis of the benefit of targeted therapies was restricted only to patients who received second-line treatment.

Time-to-event endpoints were estimated using the Kaplan–Meier method and log-rank test for statistical comparison. Cox proportional hazards models were used to obtain HRs with 95% confidence intervals (CIs). Multivariate Cox models were used to calculate HR with 95% CIs after adjusting for potential confounders (age, ECOG status, sex, primary tumor location, primary tumor resection, stage at diagnosis, and local ablative therapy). The mPFS ratios were calculated according to Mock et al., and paired comparisons were performed using the paired Wilcoxon test^20^.

The P-values were two-sided. All statistical analyses were performed using Stata software package (Stata. version 17.0; StataCorp and GraphPad Prism 9.0 (GraphPad Software. Inc. San Diego. CA).

### Consent to participate

Written informed consent for molecular analysis was obtained from each patient during the routine clinical workflow.

## Results

### Patient characteristics

**Figure 1:**
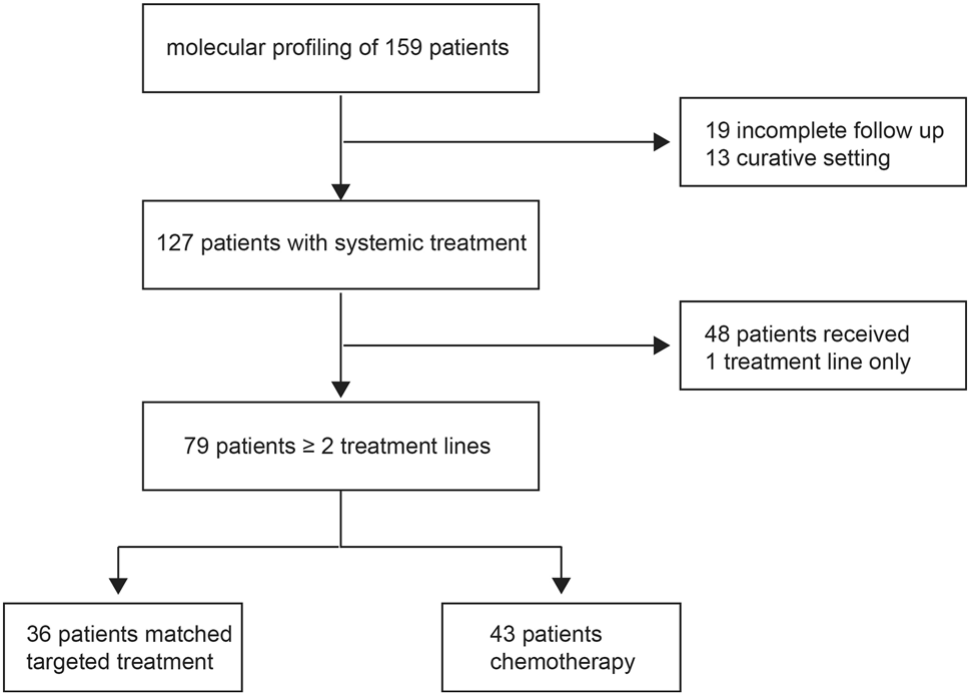
Trial design: Flow chart depicting patient allocation.

In our analysis, we included patients with biliary tract cancer and available comprehensive molecular characterization data from five Austrian cancer centers. The data cut-off date was September 2022 (Fig. 1).

In total, the molecular profile of 159 patients with biliary tract cancer was available. For further analysis, 19 patients were excluded due to incomplete clinical follow-up data, and 13 patients were excluded because they were still in the curative setting.

**Figure 2:**
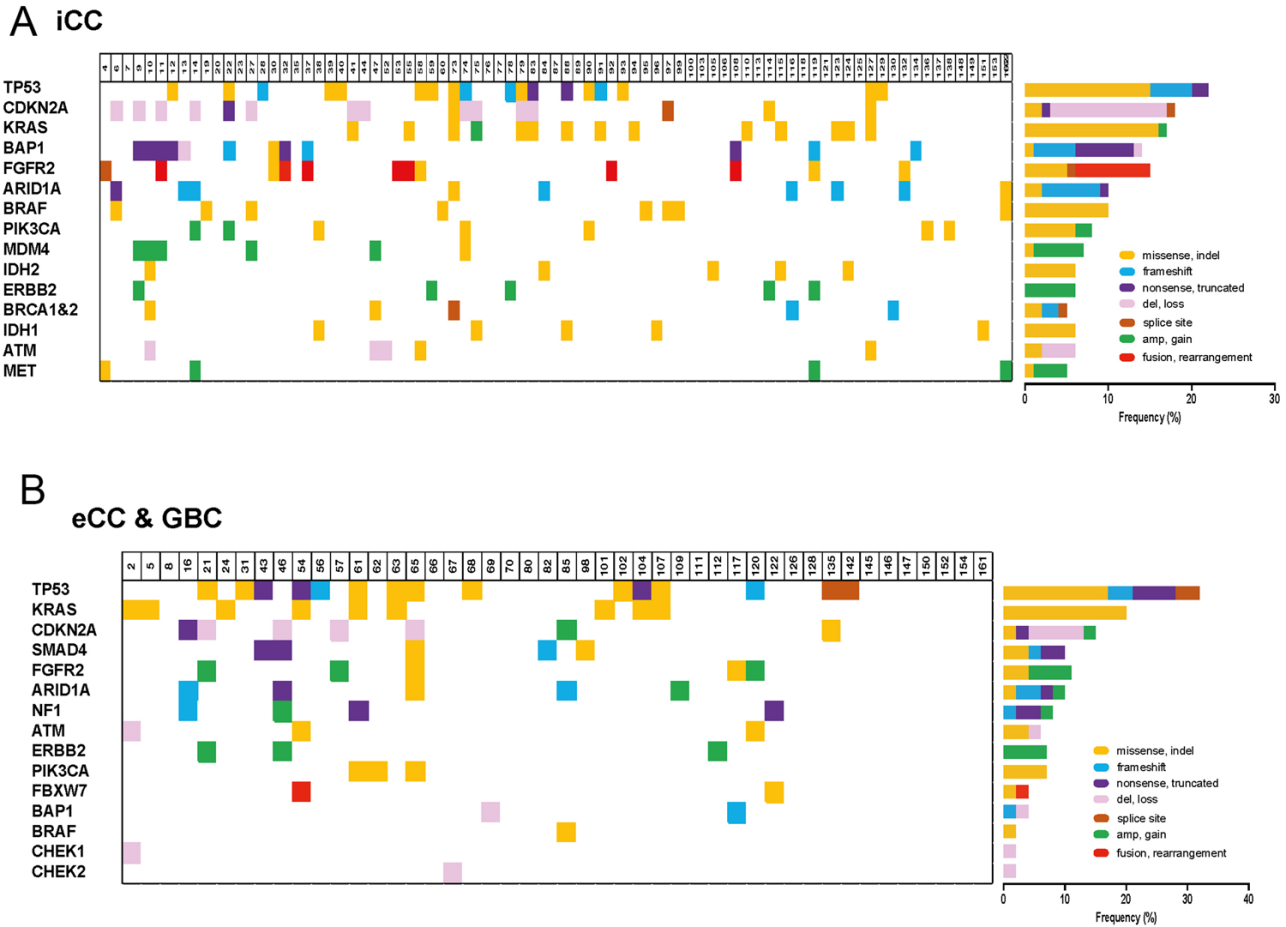
Distribution of genetic alteration according to primary tumor location.

Therefore, 127 patients received at least one line of systemic treatment. The description of the molecular landscape of extrahepatic (eCC) and gallbladder cancer (GBC) vs. intrahepatic biliary tract cancer (iCC) was based on this cohort (Fig. 2A,B).

The three most prevalent altered genes, *TP53* (32.6% for eCC vs. 22.2% for iCC; p = 0.21), *KRAS* (19.6% for eCC vs.17.3% for iCC; p = 0.81), and *CDKN2A* (15.2% for eCC vs. 18.5% for iCC; p = 0.81), were evenly distributed. Whereas in iCC alterations in genes dedicated as ESCAT I/II (*FGFR2* fusion, *BRAF-V600E* mut., *Her2neu* amplification, *IDH1* mut., MSI and *NTRK* Fusion) according to the latest ESMO guidelines for molecular testing in biliary tract cancer were more frequent than in eCC (27.5% vs. 10.5%; p = 0.0093) (Fig. 2A,B; Supplementary Table).

**Figure 3:**
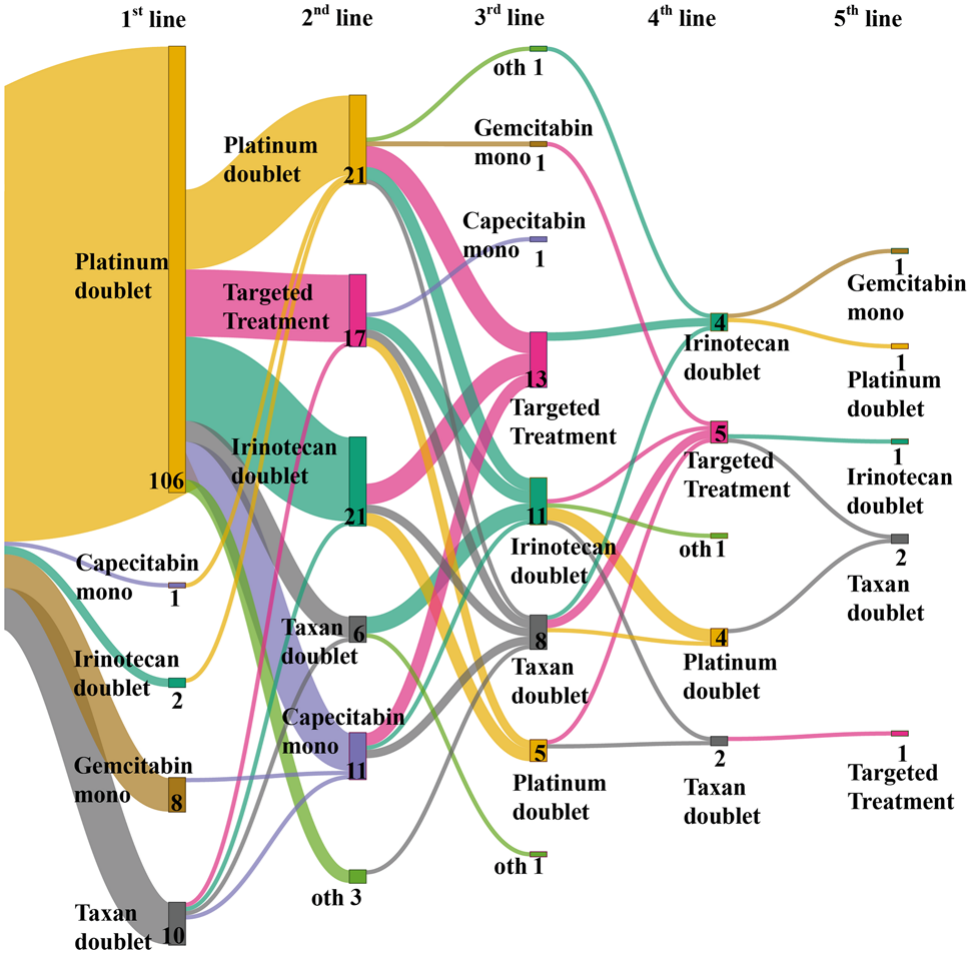
Sankey plot characterizing treatment patterns across lines of therapy.

The treatment patterns across lines of therapy are depicted in Fig. 3. 62.2% of patients with advanced unresectable or metastatic biliary tract cancer proceeded to 2nd line treatment and 32.2% received at least 3rd line therapy. Reasons not proceeding to 2nd line were in most cases deterioration of ECOG performance status (N = 22; 17.3%) or death while on first line treatment (N = 17; 13.4%).

After failure of first line strategy, 36 patients who received a matched targeted treatment beyond the first line were compared in terms of efficacy outcomes with 43 patients treated with cytotoxic chemotherapy (Fig. 1).

Patients who received targeted treatment were significantly younger than those who received chemotherapy alone; 61.9 vs. 67.6 years (p = 0.012). However, this age difference became insignificant when patients not suitable for second-line therapy were excluded (61.9 vs 64.6 years; p > 0.05). The description of the group differences between matched targeted treatment (TT) and non-targeted therapy (NTT) versus non-targeted therapy, including only patients with second-line treatment (NTT2), is depicted in Table 1. The male patient rate was consistent between the groups (TT 69.4%; NTT 56%; NTT2 65.1%; p > 0.05). The ECOG performance status was also balanced between the groups. We observed a higher proportion of iCC in the group of patients who were eligible for matched targeted treatment than in the chemotherapy group (TT 83.3% vs. NTT 56% vs. NTT2 55.8; p = 0.0041; p = 0.026). No significant difference in tumor stage distribution was detected between the two groups. Patients with targeted treatment were more often prone to primary tumor resection 41.7% vs. 33% vs. 34.9%; p = 0.412; p = 0.643) and ablative therapy (27.8% vs. 15.4% vs. 18.6%; p = 0.133; p = 0.422) during the course of the disease (Table 1).

**Table 1:** Characteristics of patients and systemic treatment lines included in the trial.

Parameters	Matched targeted treatment (TT) (n = 36) N (%)	Non-targeted therapy (NTT) (chemotherapy) (n = 91) N (%)	Non targeted therapy (NTT2) (chemotherapy) pat. with ≥ 2 lines (n = 43) N (%)
Age at diagnosis			
Median	61.9	67.6	64.6
Range	[35–81]	[38–86]	[40–86]
Sex			
W	11 (30.6%)	40 (44%)	15 (34.9%)
m	25 (69.4%)	51 (56%)	28 (65.1%)
ECOG			
0	32 (88.9%)	55 (60.4%)	30 (69.8%)
1	4 (11.1%)	27 (29.7%)	13 (30.2%)
2	0 (0%)	9 (9.9%)	0 (0%)
Localization			
iCC	30 (83.3%)	51 (56%)	25 (55.8%)
eCC	4 (11.1%	33 (36.3%)	15 (37.2%)
GBC	2 (5.6%)	7 (7.7%)	3 (7%)
Stage			
I	0 (0%)	2 (2.2%)	1 (2.3%)
II	6 (16.7%)	15 16.5%)	10 (23.2%)
III	12 (33.3%)	26 (28.6%)	7 (16.2%)
IV	18 (50%)	48 (52.7%)	25 (58.1%)
Resection primary tumor	15 (41.7%)	30 (33%)	15 (34.9%)
Local ablative therapy	10 (27.8%)	14 (15.4%)	8 (18.6%)
1st line platinum based therapy	29 (80.6%)	77 (84.6%)	39 (90.7%)
2nd line therapy			
Platinum based	7 (19.4%)	n.a.	14 (32.5%)
Irinotecan based	7 (19.4%)	n.a.	14 (32.5%)
Taxan based	1 (2.8%)	n.a.	5 (11.6%)
Capecitabin/5FU mono	3 (8.3%)	n.a.	8 (18.6%)
Matched targeted treatment	17 (47.2%)	n.a.	0 (0%)
Other	1 (2.8%)		2 (4.6%)
No of pat. with total lines of therapies			
≤ 1 L	0	48 (52.7%)	0 (0%)
≤ 2 L	10 (27.8%)	28 (30.8%)	28 (65.1%)
≤ 3 L	17 (47.2%)	8 (8.8%)	8 (18.6%)
≤ 4 L	5 (13.9%)	5 (5.5%)	5 (11.6%)
≤ 5 L	4 (11.1%)	2 (2.2%)	2 (4.7%)
Median lines of therapy	3 (2–5)	1 (1–6)	2 (2–7)
Level of evidence of matched targeted treatment			
ESCAT I/II	23 (63.7%)	n.a.	n.a.
ESCAT III/IV	13 (36.1)	n.a.	n.a.
NCT m1A/B	20 (55.6%)	n.a.	n.a.
NCT m1C	3 (8.3%)	n.a.	n.a.
NCT m2	10 (27.8)	n.a.	n.a.
NCT m3/m4	3 (8.3%)	n.a.	n.a.
Initiation of matched targeted treatment			
2nd line	17 (47.2%)	n.a.	n.a.
3rd line	13 (36.1%)	n.a.	n.a.
4th line	5 (13.9%)	n.a.	n.a.
5th line	1 (2.8%)	n.a.	n.a.

Most patients received a platinum-based combination as the first-line systemic treatment (80.6% vs. 84.6% vs. 90.7%; p = 0.602; p = 0.214). Second-line chemotherapy consisted mostly of irinotecan or platinum-based chemotherapy. In addition, in the targeted group, 47.2% of patients were already suitable for the matched targeted approach. In total, patients in the matched targeted treatment group received a median of one treatment line more (three vs. two lines; p = 0.023) than the chemotherapy group suitable for at least second-line therapy (Table 1).

### Activity of targeted treatment

In the matched targeted treatment group, the most prevalent target genes were *FGFR* (36.1%), *BRAF* (11.1%), *HER2neu* (11.1%) and MSI (11.1%). Tier I/II alterations were found most frequently in our cohort (23 patients, 63.9%). Among these, *FGFR2*-Fusion (30.4%), *BRAF V600E* mutation (13%), *Her2neu* amplification (17.4%), high PDL-1 expression (17.4%), and high MSI (17.4%) were reported. Tiers III/IV consisted of alterations in *FGFR2/3* (38.5%), *BRCA1/2* (23.1%), *PIK3CA* (15.4%), *EGFR* (15.4%), or *BRAF non V600E* (7.7%) (Fig. 2A,B; Supplementary Table).

In most patients (N = 17 or 47.2%), matched targeted treatment was initiated as second-line therapy, and 36.1% received it as 3rd line (Table 1).

**Figure 4:**
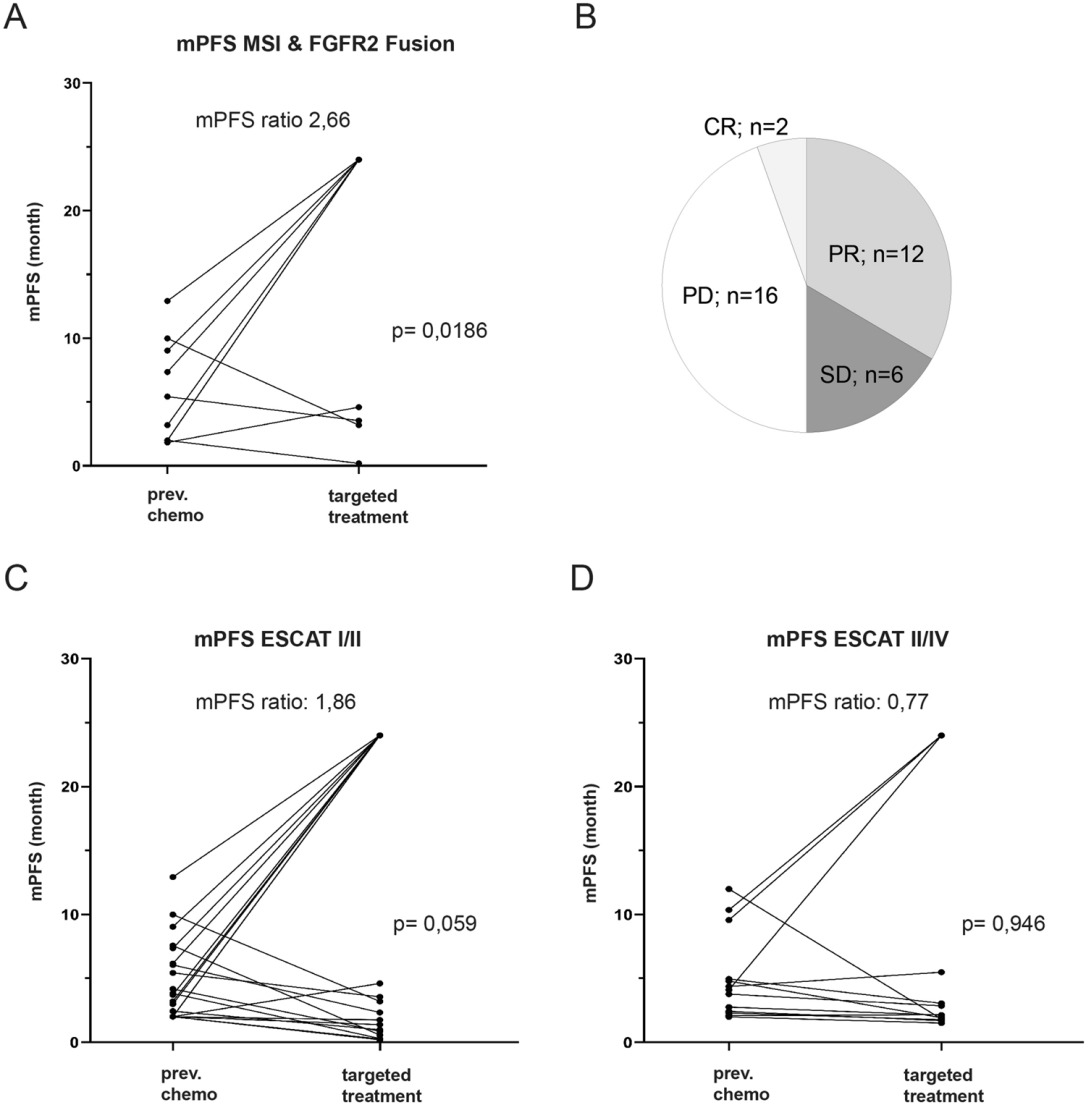
Activity of matched targeted treatment in terms of response rates and improvement of mPFS ratios. Pie chart depicting response rates of matched targeted treatment (**B**). Paired scatter plot describing mPFS of targeted treatment compared to previous chemotherapy according to ESCAT tiers (**C**,**D**) and among MSI and FGFR2 fusion patients (**A**).

The overall response (ORR) rate was 39%, with two complete responses (CR) in patients harboring MSI and 12 patients with partial remissions (PR). 6 patients (16,7%) achieved stable disease (SD). The disease control rate (DCR) defined as CR + PR + SD was 55.6% (Fig. 4B).

To further assess the clinical activity of this targeted approach, we compared the PFS of the targeted approach with that of the previous treatment. For Tier I/II alterations, we observed a modified PFS ratio (PFS_targeted_/PFS_pre-chemotherapy_) of 1.86 that was borderline significant (p = 0.059) (Fig. 4C). We further showed that FGFR2 fusion and MSI patients had the greatest benefit, with an mPFS ratio of 2.66 (p = 0.0186) (Fig. 4A). The mPFS ratio for ESCAT III/IV alterations was 0.77 (p = 0.946) with only 4 patients having a mPFS ratio greater than 1 (Fig. 4D).

### Efficacy of targeted treatment

**Figure 5:**
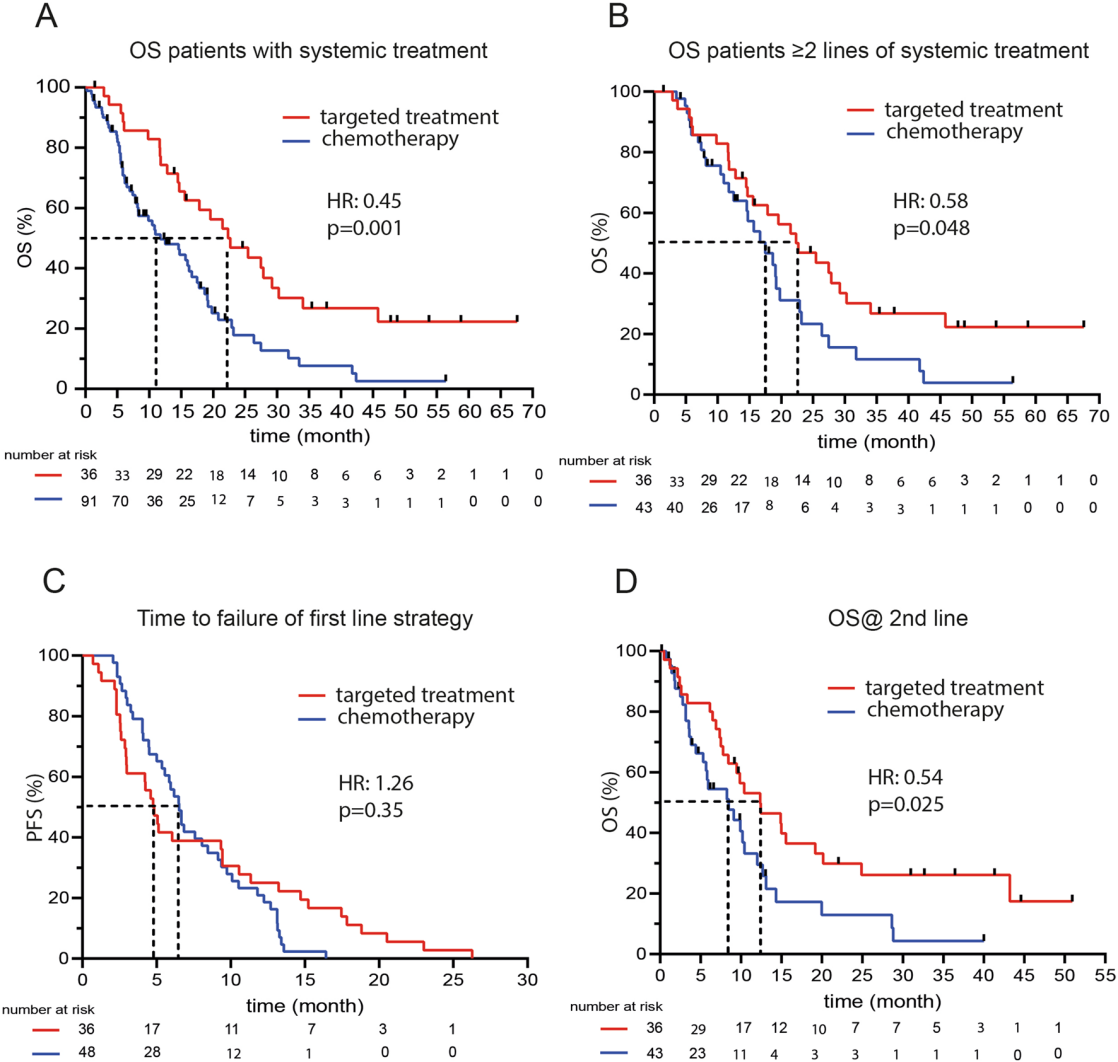
Kaplan–Meier plots comparing matched targeted treatment with chemotherapy.

Most of the patients received platinum-based first-line therapy. The time to failure of the first-line strategy was not different between the two groups and was 4.76 months (95% CI 2.926–9.436) for the targeted group vs. 6.51 months (95% CI 4.997–8.449) in the chemotherapy group (HR 1.26; 95% CI 0.777–2.038; p = 0.35; Fig. 5C).

OS in the overall cohort was 22.32 months (95% CI 14.663- 29.195) in the targeted group (N = 36) vs. 11.74 (95% CI 8.153–16.636) in the chemotherapy group (N = 91). HR of 0.45 (95% CI 0.275–0.722; p = 0.001; Fig. 5A).

Further comparison of patients who received at least two lines of therapy showed a significant OS benefit of almost 5 months for the targeted treatment group (22.32 months; 95% CI 14.663–29.195; vs. 17.49 months; 95% CI 11.74–19.79). This resulted in HR 0.58 (95% CI 0.335–0.994; p = 0.048) (Fig. 5B).

**Figure 6:**
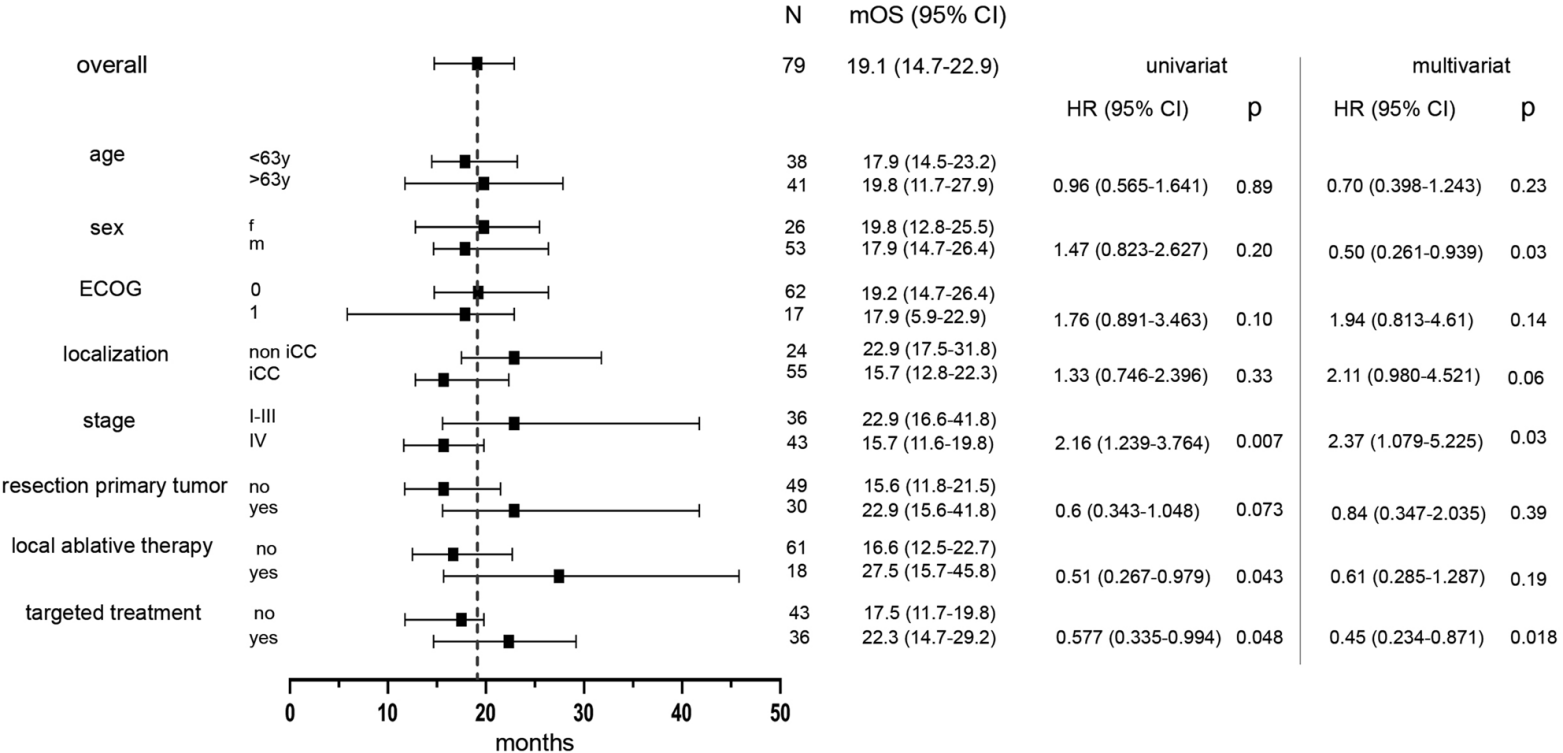
Forest plot showing potential factors with influence on outcome parameters as depicted in univariate or multivariate analysis.

After adjusting for factors such as sex, ECOG status, primary tumor resection, localization and stage in a multivariate analysis, the OS difference remained significant (p = 0.018) with a HR of 0.45 (95% CI 0.234–0.871; Fig. 6).

After the start of second-line treatment, OS was 12.36 months (95% CI 7.792–19.167) in the matched targeted treatment group and 8.48 months (95% CI 4.405–10.422) in the chemotherapy group (HR 0.54; 95% CI 0.309–0.926; p = 0.025; Fig. 5D). Statistical significance remained in the multivariate analysis (HR 0.44; 95% CI 0.229–0.854; p = 0.015).

### Efficacy of targeted treatment according to variant classification systems in precision oncology

**Figure 7:**
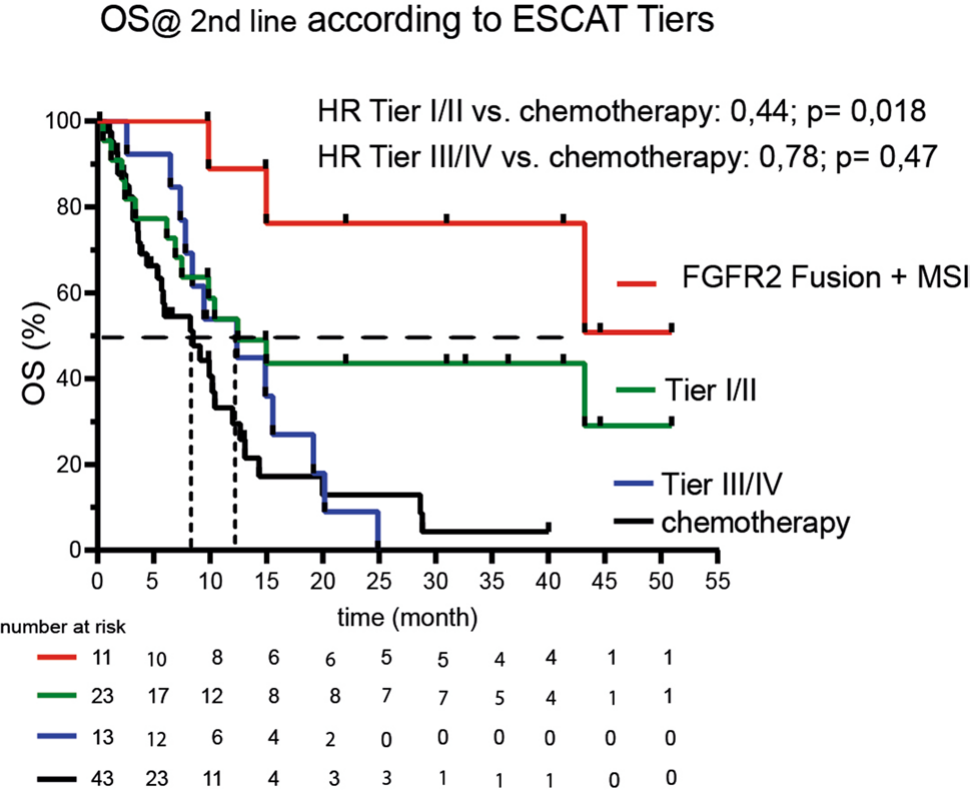
Kaplan–Meier plots of overall survival after failure of first line strategy stratified by ESCAT tiers, FGFR2 Fusion + MSI or chemotherapy.

Subdividing the targeted group according to the level of evidence for each group showed an OS benefit after first-line failure for ESCAT I/II compared to chemotherapy patients 12.43 months 95% CI 6.148–NR) vs. 8.48 months (95% CI 4.405–10.422 HR of 0.44; 95% CI 0.219–0.866; p = 0.018) (Fig. 7). Comparable results were obtained from NCT m1A/B classified variants; OS was 14.96 months 95% CI 6.15–NR) (Supplementary Figure).

For ESCAT III/IV alteration, OS was only numerically different with 12.36 months 95% CI 7.364–19.167; HR 0.78 95% CI 0.396–1.555; p = 0.47 compared to the chemotherapy group (Fig. 7).

Among ESCAT I/II alterations, *FGFR2* fusion-positive tumors treated with Pemigatinib and MSI tumors treated with checkpoint inhibitors showed the greatest benefit (N = 11; OS NR; 95% CI 9.863-NR) (Fig. 7).

## Discussion

The main focus of the trial was to demonstrate the efficacy of precision medicine in BTC in routine clinical practice, as the current evidence is predominantly derived from Asian cohorts, patients from phase 1/2 clinical trials, or distinct anatomical subtypes^13,14,23,24^. Recommendations for targeted therapies as proposed, for example, in molecular tumor boards are often hampered by missing availability of the matched treatment due to the lack of cost coverage by insurance companies, clinical deterioration during NGS turnaround times, or low evidence levels influencing the treating physician or patients^25^. In our cohort, 28% of the patients in the metastatic setting with complete molecular profiling were candidates for precision oncology. This percentage is slightly lower than the assumed 40–50% of potential exploitable targets in BTC^3^. In our setting, this could simply be due to the lack of available matched treatment for *IDH1/2*, *KRAS G12C* or *MET* amplification, as well as some missed opportunities in *FGFR2* altered or MSI high patients. The overall molecular makeup of our cohort reflected the results described in the literature. The reason for the lower frequency of *IDH1/2* mutations in our patients remains elusive.

### OS benefit of overall strategy

Our trial showed that the strategy of advanced molecular characterization in BTC almost doubled the OS compared with sequential chemotherapy. After excluding patients in the chemotherapy cohort who were not able to receive second-line treatment (due to rapid tumor progression, worsening of the ECOG performance status, decline of further cytostatic treatment, or missing target for second-line targeted treatment), we compared a more homogenous group of patients receiving at least two lines of systemic treatment. An OS benefit of 5 months was observed. As the time to failure of the first-line strategy was not different between the groups, we could clearly delineate that the OS advantage depends on the implementation of molecular informed treatments beyond the first-line. This provided a OS benefit of over 4 months when second-line treatment was initiated.

The OS time beyond failure of first-line strategy of 8.5 months was similar to results reported from prospective ABC trials or retrospective real-world cohorts^6,7,26^. Therefore, the underperformance of our control group can largely be excluded.

### Efficacy according to ESCAT classification

André et al. showed that the clinical benefit of matched targeted treatment for breast cancer seems to be confined to ESCAT I/II tiers^27^. The results from Verdaguer et al. suggest that a benefit is derived for a cohort of BTC from higher-ranked ESCAT recommendations^14^. In a subgroup analysis of our data, we can show the dominance of ESCAT I and II tiers, a result that is maintained when applying a different variant classification system (the NCT variant classifier).

The choice of a Tier I/II-matched therapy as a modified mPFS to correct for false-positive or false-negative results, as shown by Mock et al., met the threshold of 1.3, which is commonly used in precision oncology to determine the benefit of matched targeted treatment^20^. These improved PFS ratios were reflected in an OS advantage. For the subgroup of *FGFR2* Fusion or MSI patients, the mPFS ratio was even more pronounced, suggesting that OS improvement might predominantly be based on *FGFR2* Fusion and MSI patients, resulting in even more granularity in ESCTA I/II tiers.

The clinical benefits of Tiers III and IV remain controversial. Our results still advocate discussing III/IV in molecular tumor boards and applying matched therapy may be beyond second-line or searching for available clinical trials.

### Single targets

The results for MSI or *FGFR2* Fusion are in line with smaller prospective molecular-selected trials showing an OS advantage in MSI patients or *FGFR2* Fusion patients in contrast to other class I or II targets^28–30^. We cannot exclude the possibility that *FGFR2* Alterations have an additional prognostic impact, in addition to their predictive role for *FGFR2* inhibitors^31,32^.

PDL1 blockade, in addition to MSI based on PDL1 expression as a stratification marker, is a class II recommendation based on smaller phase II trials^33^. On an individual basis, we observed responses lasting up to 8 months. These results reinforce the implementation of checkpoint inhibition in CCA, with or without chemotherapy. In contrast, we did not find targeting *Her2neu* with Trastuzumab +/− Pertuzumab despite promising phase II data^34^. All four patients showed tumor progression. One reason might be that different co-mutations affect the clinical outcome parameters. However, according to the data from ASCO 2022 and reports from other tumor types, targeting *HER2neu* with Trastuzumab-deruxtecan would probably yield better results^35^.

Unfortunately, blocking of *IDH1* was not available in Europe. Access to *IDH1* inhibitors, such as Ivosidenib, will further expand the therapeutic options and may further improve the clinical benefit for patients with BTC.

### Limitations

A drawback of our trial was that MSI and TMB testing was not performed routinely in every patient; thus, patients who may have been candidates for checkpoint inhibitors were potentially missed. Furthermore, the extent of the NGS panel was not consistent in our patients. This reflects the diagnostic advances in molecular techniques over time and the different availability of NGS panel diagnostics in various centers.

Further limitations are due to the retrospective nature of our analysis and, presumably, tumor spatial and temporal heterogeneity. Ct-DNA isolated from blood or bile samples could overcome these limitations.

## Summary

Our results underscore the value of comprehensive molecular testing for BTC and reinforce the statement of the ESMO guidelines that every patient with BTC should undergo molecular characterization. We clearly showed that, in contrast to unmatched cytotoxic chemotherapy, the application of molecular matched targeted treatment leads to an OS benefit in a real-world setting. Further research must focus on the resistance mechanisms of the targeted agents and how to overcome these limitations.

### Supplementary Information

Supplementary Legends.

Supplementary Figure 1.

Supplementary Table 1.

## Data Availability

The datasets used and/or analysed during the current study are available from the corresponding author on reasonable request.
