# Supplementary material for: Real world evidence reveals improved survival outcomes in biliary tract cancer through molecular matched targeted treatment
Source: Sci Rep. 2023 Sep 18;13:15421. doi: 10.1038/s41598-023-42083-4 (PMC10507096; doi:10.1038/s41598-023-42083-4)
Supplement: Supplementary file 1 — Supplementary Legends. [file 41598_2023_42083_MOESM1_ESM.docx]

Supplement

**Legends**

Supplementary Table: Molecular matched targeted treatment and corresponding clinical efficacy at the individual patient level

Supplementary Figure: Kaplan-Meier plots of overall survival after failure of the first-line strategy stratified by the NCT variant classification system
