# Supplementary figures and images for: Real world evidence reveals improved survival outcomes in biliary tract cancer through molecular matched targeted treatment
Source: Sci Rep. 2023 Sep 18;13:15421. doi: 10.1038/s41598-023-42083-4 (PMC10507096; doi:10.1038/s41598-023-42083-4)

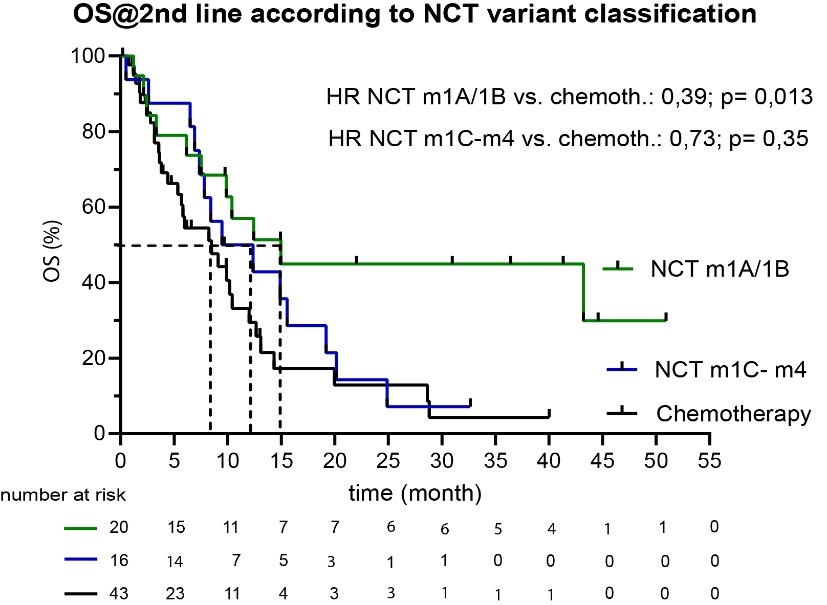

Supplement: Supplementary file 2 — Supplementary Figure 1. [file 41598_2023_42083_MOESM2_ESM.jpg]
