# Supplementary material for: Real world evidence reveals improved survival outcomes in biliary tract cancer through molecular matched targeted treatment
Source: Sci Rep. 2023 Sep 18;13:15421. doi: 10.1038/s41598-023-42083-4 (PMC10507096; doi:10.1038/s41598-023-42083-4)
Supplement: Supplementary file 3 — Supplementary Table 1. [file 41598_2023_42083_MOESM3_ESM.docx]

**Supplementary Table1**

| Pat ID | Molecular target | Line of therapy | Molecular informed therapy | ESCAT level | NCT  Variant  Classification | OS  (month) | Best overall response |
| --- | --- | --- | --- | --- | --- | --- | --- |
| 4 | FGFR2::CENPE | 2nd | Pemigatinib | I-B | m1A | 68 | PR |
| 11 | FGFR2::WAC | 3rd | Pemigatinib | I-B | m1A | 54 | PR |
| 32 | FGFR2::HOOK1 | 3rd | Pemigatinib | I-B | m1A | 20 | SD |
| 37 | FGFR2::BICC1 | 2nd | Pemigatinib | I-B | m1A | 25 | PR |
| 53 | FGFR2::KIAA1217 | 2nd | Pemigatinib | I-B | m1A | 13 | PR |
| 55 | FGFR2::NN | 2nd | Pemigatinib | I-B | m1A | 16 | PR |
| 108 | FGFR2::OFD1 | 4th | Pemigatinib | I-B | m1A | 46 | PR |
| 30 | FGFR2 p.S373C | 3rd | Pemigatinib | I-C | m1C | 38 | PR |
| 16 | MSH6 p.F1104Lfs*11 | 3rd | Nivolumab | I-C | m1B | 58 | PR |
| 61 | MSI- IHC | 2nd | Pembrolizumab | I-C | m1A | 2 | PD |
| 28 | MSH6 p.F1088Sfs*2 | 2nd | Pembrolizumab | I-C | m1A | 35 | PR |
| 123 | MSH6 p.R248Tfs*8 | 3rd | Pembrolizumab | I-C | m1A | 49 | PR |
| 27 | BRAF p.V600E | 2nd | Dabrafenib/Trametinib | I-B | m1A | 3 | PD |
| 162 | BRAF p.V600E | 3rd | Dabrafenib/Trametinib | I-B | m1A | 15 | PR |
| 95 | BRAF p.V600E | 3rd | Dabrafenib/Trametinib | I-B | m1A | 10 | PR |
| 87 | HER2neu 3+ | 3rd | Trastuzumab/Pertuzumab | I-C | m1A | 16 | PD |
| 59 | HER2neu 3+ | 2nd | Trastuzumab/Pertuzumab | I-C | m1A | 6 | PD |
| 112 | HER2neu 3+ | 2nd | Trastuzumab | I-C | m1C | 6 | PD |
| 78 | HER2neu 3+ | 2nd | Trastuzumab | I-C | m1C | 12 | PD |
| 60 | PDL-1 90% | 2nd | Pembrolizumab | II-A | m1B | 6 | PD |
| 39 | CPS 90% | 2nd | Nivolumab | II-A | m1B | 12 | PD |
| 66 | TPS 90% | 3rd | Nivolumab | II-A | m1B | 29 | PD |
| 43 | TPS 90% | 4th | Nivolumab | II-A | m1B | 48 | PR |
| 38 | PIK3CA p.H1047R | 2nd | Alpelisib | III-A | m2A | 4 | PD |
| 47 | BRCA1 p.C61G | 2nd | Olaparib | III-A | m2A | 14 | PD |
| 10 | BRCA2 p.R2784W | 4th | Olaparib | III-A | m2A | 28 | SD |
| 130 | BRCA2 p.S2186fs | 2nd | Olaparib | III-A | m2A | 12 | SD |
| 96 | EGFR amp. | 2nd | Cetuximab | III-A | m2B | 26 | PR |
| 97 | EGFR amp. | 2nd | Cetuximab | III-A | m2B | 34 | PD |
| 35 | FGFR3::TACC | 3rd | Pemigatinib | III-B/IV | m3/m4 | 23 | SD |
| 57 | FGFR amp. | 3rd | Temsirolimus | III-B/IV/V | m4 | 18 | PD |
| 58 | FGFR2 p.E565G | 5th | Pazopanib | III-B/IV/V | m2B | 30 | PD |
| 132 | FGFR2 p.N549K | 3rd | Regorafenib | III-B/IV/V | m2B | 28 | SD |
| 9 | FGFR3 amp. | 4th | Regorafenib | III-B/IV/V | m2B | 15 | PD |
| 90 | PIK3CA p.N1044K | 3th | Regorafenib | III-B/IV/V | m2B | 22 | PD |
| 99 | BRAF p.G466V | 4th | Everolimus | IV | m4 | 22 | SD |
